# Views of Consumers, Farmers and Stakeholders on Alternative Dairy Cattle Housing Systems

**DOI:** 10.3390/ani12101231

**Published:** 2022-05-10

**Authors:** Karmen Erjavec, Marija Klopčič

**Affiliations:** 1 Faculty of Economic and Informatics, University of Novo Mesto, Na Loko 2, 8000 Novo Mesto, Slovenia; karmen.erjavec@uni-nm.si; 2 Biotechnical Faculty, University of Ljubljana, Jamnikarjeva 101, 1000 Ljubljana, Slovenia

**Keywords:** housing system, dairy cattle, attitudes, acceptability

## Abstract

**Simple Summary:**

Sustainable alternative housing systems for dairy cattle depend on being accepted by key categories like farmers, stakeholders, and consumers. Alongside tie-stall and cubicle housing systems, alternative free-walk systems (compost-bedded pack and artificial floor systems) are available today. This study aimed to determine and compare the acceptance of (alternative) housing systems and attitudes to certain aspects of housing systems for dairy cattle among Slovenian consumers, farmers, and stakeholders. They were asked online about their views on the most important aspects (animals, farmers, products, environment) of housing systems as well as the acceptance of four housing systems and related issues. The results reveal differences in attitude and acceptance among the main *groups* of respondents. Consumers, stakeholders, and conventional farmers preferred the artificial floor system, while organic farmers preferred the compost-bedded pack housing system. Consumers and organic farmers showed below-average scores for all aspects analyzed concerning animals, products, and the environment, whereas conventional farmers and stakeholders had shown above-average scores for aspects related to animals and the environment and negative attitudes to products. The findings suggest the need to tailor information about different housing systems to specific groups.

**Abstract:**

Alternative housing systems for dairy cattle have recently emerged, such as compost-bedded packs and artificial floor ones. To determine their acceptability among categories of people with a connection to animal husbandry, this study aimed to identify and compare the acceptability of (alternative) housing systems and attitudes to aspects of housing systems for dairy cattle among Slovenian consumers, farmers, and stakeholders. Farmers (N = 306), consumers (N = 508), and stakeholders (N = 40) were interviewed about their views on the main aspects (animals, farmers, products, environment) of housing systems for dairy cattle, the acceptance of four housing systems, and important housing features. The results show that consumers, stakeholders, and conventional farmers preferred housing systems with an artificial floor, while organic farmers preferred a housing system with a compost-bedded pack. Consumers and organic farmers expressed the greatest acceptance of almost every aspect of the housing system, except for a sufficient income for farmers and a low workload. Conventional farmers and stakeholders hold similar views, except for the expectation that the animals have enough space to move around, the image of the landscape, and the animals’ health and wellbeing, where stakeholders showed more acceptance than conventional farmers. The results imply that systematically planned information aimed at different target categories is needed to increase the acceptance of (alternative) housing systems for cattle.

## 1. Introduction

**Figure 1:**
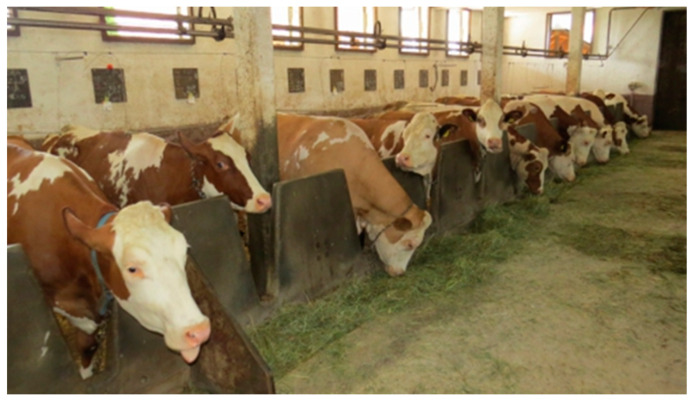
Tie-stall barn.

**Figure 2:**
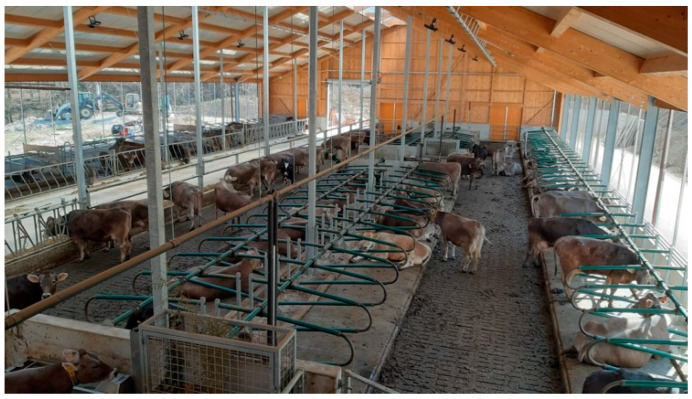
Freestall barn with cubicles.

**Figure 3:**
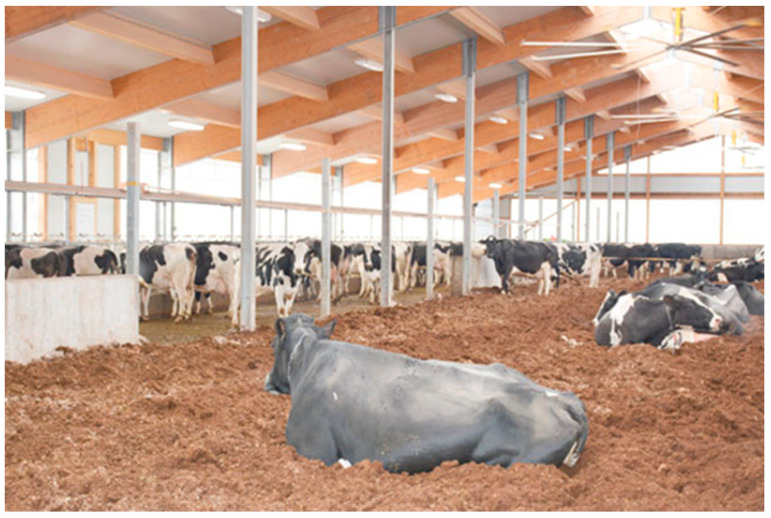
Compost-bedded pack barn.

**Figure 4:**
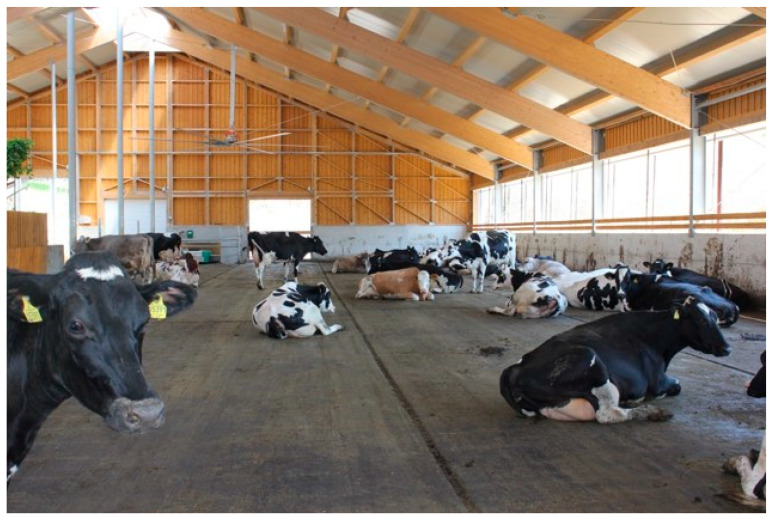
Free-walk housing system with artificial floor.

In recent years, a few alternative housing systems for dairy cattle have emerged, generating discussion among the professional and lay public and the media. Alongside common housing systems like the tie-stall (Figure 1) and the cubicle (also called free stall) (Figure 2), alternative free walk housing systems, i.e., compost-bedded pack (Figure 3) and artificial floor systems (Figure 4), are growing in popularity not only in the countries where they were initially developed such as Israel and the USA but also with some modification in Europe and less developed dairy countries like Slovenia [1,2,3,4,5,6]. Available data indicate that free walk housing systems increase cow welfare by providing more space and allowing animals to rest without being restricted by cubicle partitions as occurs with tie-stalls and cubicle housing systems [1,2,3,7].

Attitudes to a housing system, simply defined as evaluations of it containing affective, behavioral, and cognitive components [8], differ among categories of people who are connected to animal husbandry [4,5,6]. For example, a study of consumers’ attitudes toward tie-stall, cubicle, compost-bedded, and artificial floor housing systems in Austria, Germany, Italy, the Netherlands, Norway, Slovakia, Slovenia, and Sweden showed the compost-bedded housing system was liked the best in all countries and is associated with better animal welfare, followed by the artificial floor, cubicle, and the tie-stall [5]. However, practices like organic farming and cow grazing have been shown to be more important for consumer willingness to pay for products from the farm than the particular housing system. Indeed, many studies show that consumers value cows having the ability to graze or being in an environment that mimics the outdoors [5,9,10,11,12,13,14]. A recent analysis of German citizens’ public acceptance of four common housing systems for dairy cattle confirms that the lack of cows’ possibility to behave naturally was the key reason for housing systems’ low acceptance rates [10].

Farmers have the greatest influence on the adoption of alternative housing systems. A study on innovation in agriculture found that most farmers hold a cautiously positive attitude to innovation generally, reflecting a pragmatic approach to business survival, regardless of the characteristics of the farm and the farmer [15]. Still, the majority of them are not concerned by the multifaceted crisis of agriculture-related to its influence on the climate crisis and loss of biodiversity [16,17,18,19]. Conventional farmers’ primary motives for adopting an animal and environmentally friendly housing systems for dairy cattle are to achieve higher production and profits and to receive full direct payments [20,21]. A study on farmers’ opinions regarding cubicle and compost-bedded housing systems in Austria, Germany, Italy, the Netherlands, Slovenia, and Sweden found that farmers judged the compost bedded-pack system (CBS) as more sustainable than the cubicle housing system [6]. However, they believed the cost of bedding material was a serious drawback of this system.

Others with an influence on alternative housing systems for dairy cattle are stakeholders like policy makers, agricultural advisors, nutritionists, herd veterinarians, and animal scientists. Their important role makes it surprising that only a few studies have presented their views, and none seem to have investigated their attitudes to housing systems for dairy cattle. A study on views about the ideal dairy farm in which agricultural advisors also participated showed that in contrast to lay citizens, who attributed the greatest value to the quality and naturalness of milk, both farmers and advisors stressed profitability and biological functioning as the most important characteristics of the ideal farm, including its housing system [13]. A study of Dutch advisors’ views on pig husbandry reveals they hold attitudes similar to conventional pig farmers, as opposed to the other category including citizens and organic pig farmers [22].

Given that the active involvement of various stakeholders to determine attitudes to animal husbandry is important for identifying animal husbandry practices that are sustainable [23,24], it is vital to detect their views on housing systems, especially novel or alternative ones. Since existing studies on housing systems for dairy cattle have not investigated alternative housing systems’ acceptability among three key interesting categories—Farmers, consumers, and stakeholders—This study aimed to fill a research gap and answer the research question: Do differences exist among farmers, consumers, and stakeholders regarding the acceptability of (alternative) housing systems for dairy cattle and attitudes to different housing system aspects in typical Central European countries like Slovenia? The study aimed to identify and compare the acceptance of (alternative) housing systems and attitudes concerning different aspects of housing systems for dairy cattle among Slovenian farmers, stakeholders, and consumers in general. Furthermore, the utilitarian aim is to determine which aspects should be included in the planned communication for different groups or categories to increase social acceptance of the introduction of a sustainable alternative housing system.

## 2. Materials and Methods

### 2.1. Participants

On 1 November 2020, farmers were invited with a link to an online survey sent by e-mail to all cattle-breeding associations and categories of cattle breeders (around 1000 e-mail addresses) and via a social network (Facebook) to various associations and categories of cattle breeders in Slovenia. The survey remained active for 14 days and potential respondents were twice invited to participate.

The survey attracted 306 respondents, including 80.7% (*n* = 247) conventional farmers and 19.3% (*n* = 59) organic farmers, whose socio-demographic characteristics represent the situation of both farmers and agriculture in Slovenia [25]. In terms of gender, 75.2% of respondents were male among conventional farmers, 71.0% among organic farmers, 75.6% among stakeholders, and 53.7% among consumers. The majority of conventional farmers (33.7%) were between 36 and 45 years of age, the majority of organic farmers (39.1%) were of similar age, the majority of stakeholders (51.5%) were between 46 and 55 years of age and the majority of consumers (33.5%) were over 56 years of age. The majority of conventional farmers (45.4%) had completed secondary school, the majority of organic farmers (56.7%) and stakeholders (74.3%) had a university degree (bachelor’s) and the majority of consumers (57.3%) had completed secondary school (see Table 1).

The views of Slovenian consumers were obtained in May 2021 by a market research company through an online survey. Table 1 presents the socio-demographic characteristics of the nationally representative sample of consumers (N = 508).

Agricultural stakeholders were interviewed between 15 June and 23 July 2021. Due to the poor respondent rate in the pilot study, and limited time and motivation, stakeholders completed the surveys personally online via Teams MS, telephone, or by a face-to-face interview, according to the participants’ choice. Table 1 shows the characteristics of the sample of stakeholders (N = 40), made up of agricultural extension agents, veterinarians, input suppliers, representatives of the dairy industry, agricultural cooperatives, farmers’ associations/unions, policymakers (representatives of the Ministry of Agriculture) and experts (professors and researchers from university/research institutes).

### 2.2. Survey Design

The same questionnaire was used by all respondent categories. Each participant was asked to provide their socio-demographic characteristics (gender, age, education). The questionnaire on attitudes to specific different housing systems and the 13-item questionnaire on acceptance of aspects of the housing system, in general, was adopted from previous studies on [5,10,25], including different aspects: Animals (health, welfare, shelter), Farmers (income, work), Product (price, taste, health) and Environment (environmental impact, image landscape).

To determine the level of acceptance of alternative housing systems, the two housing systems for dairy cows commonly used in Slovenia (tie-stall, cubicle barn) together with two alternative housing systems (compost-bedded and artificial floor) were presented. To ensure all participants possessed the same level of knowledge while evaluating the housing systems, they were presented with realistic pictures and a brief neutral description of each system. The level of acceptability was measured on a 5-point scale ranging from 1 (totally unacceptable) to 5 (totally acceptable).

A pre-test was conducted with housing system experts (N = 6) and conventional and organic farmers (N = 20) and consumers (N = 20). Photos and descriptions of the housing system were adapted several times to make them understandable for consumers. Cronbach’s coefficient α was used to calculate the internal consistency coefficients of the questionnaire items. Results of the reliability analysis revealed the items held satisfactory discriminatory power in the scales of acceptability and attitudes given that α exceeded 0.75 for all items.

### 2.3. Data Analysis

Descriptive statistical analysis was conducted to determine the acceptance and attitudes of consumers, conventional dairy farmers, organic dairy farmers, and stakeholders to the housing/husbandry systems. The probability that respondents belonging to a certain respondent category gave higher or lower acceptance levels than respondents in the other categories was calculated using ordered multinomial logistic regression. A correlation was made for socio-demographic features to ensure they did not affect probabilities. Descriptive statistical analysis (multi-criteria ANOVA with a Bonferroni inequality approach) was performed to determine the acceptability of housing systems and attitudes to aspects of husbandry in the respondent categories “consumers”, “conventional farmers”, “organic farmers” and “stakeholders”. For the statistical analysis, IBM SPSS Statistics 24 was used.

## 3. Results

The results reveal (see Table 2) statistically significant differences in the level of housing systems’ acceptance by respondent categories. Housing systems with an artificial floor were considered to be the most acceptable housing system by consumers, followed by stakeholders and conventional farmers, and the least acceptable by organic farmers. The compost-bedded housing system was considered to be the most acceptable housing system by consumers, then by organic farmers, and stakeholders, and the least acceptable by conventional farmers. Both the tie-stall and cubicle housing were accepted the most strongly by conventional farmers, followed by stakeholders, organic farmers, and consumers.

The four categories of respondents had varying levels of acceptance of different aspects of the housing systems in general (Table 3). Consumers and organic farmers expressed the strongest acceptance for almost all aspects, except for a sufficient income for farmers and a low workload. Although both categories rated acceptance higher than the average and did not differ statistically significantly, consumers attributed higher values than organic farmers concerning two aspects: good animal health and the possibility for animals to go outdoors, and healthy products. For these aspects, consumers also had the highest levels of acceptance (≥3.9) among the respondent categories.

Compared to organic farmers, conventional farmers had a lower level of acceptance for most housing system aspects, except for greater acceptance (≥4.1) with respect to enough income for farmers and their low workload. The probability that stakeholders had a lower level of acceptance than conventional farmers was statistically significant concerning just three aspects: enough space for animals to move, the image of the landscape, and good animal health.

## 4. Discussion

This study is the first to compare the views of different categories of housing systems and aspects associated with alternative dairy cattle housing systems, i.e., animals, farmers, products, and the environment. We adapted a questionnaire used in previous studies [5,10,22] to make the questionnaire suitable for all categories of respondents and to focus on aspects that had triggered professional and public/media discussion. Acceptance levels of housing systems provide information on which housing systems are generally more likely to be accepted, whereas attitudes show which aspects of housing systems should be focused on while developing policies for sustainable alternative housing systems for dairy cattle. This is vital because its long-term sustainability cannot be guaranteed without the key aspects of an alternative housing system being accepted by all relevant groups in society [22,23,24,26]. The results thus indicate which alternative housing systems for dairy cattle are suitable for wider adoption in agriculture. They also suggest which aspects of current and future cattle housing systems are seen as low in importance, but should still be improved, such as the environmental aspect.

In line with other studies, Slovenian respondents evaluate the housing system with a tie-stall the worst [6,10], with large statistically significant differences among the systems. Consumers, followed by organic farmers and stakeholders, gave the lowest score, while conventional farmers gave an above-average score to this housing system. This may be explained by the fact that over 50% of Slovenian conventional dairy farmers still use this housing system, similar to other Eastern European countries [24]. Cubicle housing was the most accepted by conventional farmers, which is unsurprising given that it is the dominant housing system on European dairy farms.

In contrast to our study, which showed that all respondent categories (except organic farmers) preferred the alternative housing system with an artificial floor, a study conducted in Austria, Germany, Italy, the Netherlands, Norway, Slovakia, Sweden, and also in Slovenia revealed that consumers preferred the compost bedded-pack system over the artificial floor [5,6]. What can explain these different results? One explanation is that the housing system with an artificial floor was recently introduced into Slovenia by the conventional dairy farmer, and also has been positively and widely presented by the media as an environment that mimics the outdoors grazing situation [27].

Yet, why did organic farmers evaluate the acceptability of a housing system with an artificial floor lower than a compost-bedded pack system? Perhaps the reason is that the ‘artificial’ floor looks less natural than a compost-bedded pack, which relies on the composting of the bedding materials and a biological process. Naturalness and environmentally friendly farming are viewed as more important by organic farmers than by conventional farmers [13].

Significant differences between respondent categories in attitudes and acceptance of aspects of housing systems suggest a division into two distinct sides. One group consists of consumers and organic farmers who indicated the highest level of acceptance for almost all aspects, except for a sufficient income for farmers and a low workload. The second group consists of conventional farmers and stakeholders who held a positive attitude toward aspects related to animals and the environment. The study also confirmed findings of other studies that showed conventional farmers and stakeholders are very concerned about the quality of life, especially about workload [13,16,17,20]. Still, this is not surprising since overwork and physical load continue to be the greatest worry for farmers [28]. This can also be partly explained by the fact that productive, economic, and social conditions determine young people’s willingness to take up farming [13].

The results confirm the findings of a Dutch study on attitudes regarding pig husbandry showing that conventional pig farmers and citizens hold similar attitudes, in contrast to citizens and organic pig farmers [22]. As suggested, these differences or similarities can be explained by different interests [22,29]. Consumers are interested in animal welfare, the quality of milk and dairy products, and low prices [5,11,12,13,14], organic farmers are interested in animal health and welfare, the quality of dairy products, and the environment [30,31], while conventional farmers have a stronger interest in the economic results. The shared interest in animal welfare and the environment [32] is a key reason that consumers and organic farmers hold a similar view on housing systems.

Consumers hold negative attitudes toward housing aspects because they believe that animals have a value and feel negative emotions when treated badly or not in a manner appropriate to the species [22], or because they are influenced by advertising images largely portraying cows as happy when in the pasture [10]. However, their cultural/physical distance from the typical farm coupled with their lack of knowledge [27,33] prevents them from having a clear opinion on all issues.

Conventional farmers and stakeholders evaluated cow housing systems from the perspective of entrepreneurs. Although conventional farmers consider the environment and natural resources as important elements of their daily work, while ethical and social values are also important to them [31], they are profit-driven (i.e., the environment is justified by ethical, social, and ecological values, but requires economic sacrifices) and are willing to introduce changes and implement innovations if they expect positive economic results and/or receive full direct payments [20]. Thus, conventional farmers would consider the environment when (economic) initiatives are in place to do so [28]. Conventional farmers ranked climate change below food security, energy security, and water quality in terms of important issues facing society [30]. While organic farmers also have economic interests, environmental, ethical, and social values dominate because they have a more complex and philosophical approach to farming [31]. The results show that consumers and organic farmers have the same perspective on alternative housing systems. For both categories, aspects related to animals, products, and the environment are very important, based on interests, emotions, and the available (lack of) know-how [10,27,31].

The results also show that views do not differ according to the socio-demographic characteristics of the respondents, which is also in line with the results of a Dutch study on pig husbandry [22].

In Slovenia and elsewhere in Europe different views on housing systems between categories of respondents can conflict [20,33]. To make consumers understand the attitudes of conventional farmers, they must focus on those aspects where attitudes differ between them and consumers, i.e., the health and welfare of animals, the environment, and economics.

It would be beneficial for conventional farmers to consider the consumer’s perspective, paying special attention to animal welfare, the environment, and price. Agricultural policymakers would benefit from including and highlighting animal welfare measures to justify financial support for alternative housing systems. Since Slovenian consumers are no different from other consumers and strongly prefer housing systems with paddocks and pasture [20], but at the same time hold limited knowledge about housing systems and the agricultural situation in Slovenia, where little grazing occurs on pasture [34], they would benefit from being informed about a key element of the housing system and the agricultural sector’s efforts to improve the housing system for dairy cows and young stock. However, farmers with an opportunity to introduce housing systems with paddocks would benefit from integrating systems since these are valued by consumers. It would also be beneficial if basic information about housing systems and efforts to introduce alternative systems were not paternalistic, strongly persuasive, or overly technical. Instead, they could be emotional and inclusive and convey a good feeling and sense of considering accepting the benefits of an alternative system. Easily understood and inclusive communication is useful while discussing issues that come into conflict [35]. Although consumers rated the housing system with an artificial floor relatively high, one may question whether housing systems that provide no access to the outdoors can convince most consumers.

The ongoing COVID-19 pandemic together with the more difficult access to the stakeholders means the group of stakeholders may have been small, and data were collected in person through interviews rather than using the same technique (online) as with the consumers and farmers. Still, this was the only way of obtaining data from stakeholders in a relatively complex situation. Future studies could therefore collect data for all respondents in the group using the same technique and on a larger scale. Since we were unable to reach as many different stakeholder categories to establish their potentially different attitudes, and we assume that views among stakeholders are as diverse as among farmers, we suggest that future studies also examine the specific types of stakeholder categories. Future studies could be extended to another social group, namely students. Perhaps the problem of a limited number of stakeholders could be solved by involving students, as they are one of the important links responsible for implementing modern solutions in animal production, including dairy farming [36].

## 5. Conclusions

Views on housing systems differ among consumers, organic and conventional farmers, and stakeholders. Consumers, stakeholders, and conventional farmers prefer the housing system with an artificial floor, yet organic farmers prefer the compost-bedded pack housing system.

Significant differences between respondent categories in their level of acceptance of housing system aspects suggest a division into two distinct sides. One side consists of consumers and organic farmers who expressed the strongest acceptance of almost every aspect of the housing system (animal health, animal welfare, quality of milk and dairy products, the environment), except for a sufficient income for farmers and a low workload. The second group consists of conventional farmers and stakeholders who indicated the same level of acceptance, except for the expectation that the animals have enough space to move around, the image of the landscape, and the good health of the animals, where stakeholders showed greater acceptance than conventional farmers.

As society-wide acceptance is essential for the introduction and maintenance of a sustainable alternative housing system, systematically planned communication targeting the various groups involved on-farm and off-farm can help to increase the acceptance of alternative housing systems for cattle, thereby improving animal welfare and reducing their environmental impact. The remarkably high level of appreciation in Slovenia for the very alternative artificial floor system illustrates this. Intensive country-wide communication, including television broadcasting about this system, has led within a few years to this system being viewed positively.

## Figures and Tables

**Table 1:** Percentage of respondents per socio-demographic category for each category of respondent.

Socio-Demographic Feature	Category	Conventional Farmers (N = 247)	Organic Farmers (N = 59)	Stakeholders (N = 40)	Consumers (N = 508)
Gender	Male	75.2	71.0	75.6	53.7
	Female	24.8	29.0	24.4	46.3
Age	˂25	22.2	21.3	-	3.9
	26–35	20.9	21.9	-	13.4
	36–45	33.7	39.1	21.3	23.6
	46–55	13.7	12.1	41.5	25.6
	>56	9.5	5.6	37.2	33.5
Education	Primary school	5.2	0	-	2.2
	Secondary school	45.4	32.7	2.5	57.3
	University undergraduate	41.3	56.7	74.3	34.4
	Postgraduate	-	8.1	23.2	6.1

**Table 2:** Mean scores and standard deviations of housing systems’ acceptability per respondent category.

Housing System	Conventional Farmers $\bar{x}$ (SD)	Organic Farmers $\bar{x}$ (SD)	Stakeholders $\bar{x}$ (SD)	Consumers $\bar{x}$ (SD)	F-Value
Tie-stall	3.3 (1.1)	2.2 (1.2)	2.5 (1.3)	2.0 (0.9)	8.8 *
Cubicle	3.6 (0.8)	3.3 (0.9)	3.5 (0.9)	3.1 (0.7)	7.1 *
Compost-bedded	3.7 (1.1)	3.9 (1.2)	3.8 (1.3)	4.0 (0.8)	8.6 *
Artificial floor	3.8 (0.9)	3.5 (0.9)	3.9 (0.8)	4.1 (0.7)	9.9 *

* The mean difference is significant at *p* < 0.05, multi-criteria ANOVA with a Bonferroni inequality approach.

**Table 3:** The average level of acceptance on a 5-point scale per housing system aspect per respondent category.

Entity	Aspect	Conventional Farmers	Organic Farmers	Stakeholders	Consumers
Animal health	Good health	3.3 ^c^	3.8 ^a^	2.9 ^d^	3.9 ^a^
Animal welfare	Feel good	2.7 ^b,c^	4.2 ^a^	2.8 ^b,c^	3.9 ^a^
	Natural behavior	3.3 ^b^	4.6 ^a^	3.4 ^b^	4.3 ^a^
	Possibility to go outside	1.6 ^b^	3.9 ^a^	1.5 ^b^	4.1 ^a^
	Enough space to move	2.4 ^c^	4.1 ^a^	2.1 ^d^	3.9 ^a^
Shelter	Protection from climatic conditions	3.5 ^c^	3.8 ^a^	3.6 ^c^	3.6 ^a^
Farmer	Enough income	4.6 ^b^	4.4 ^b^	4.5 ^b^	3.7 ^a^
	Low workload	4.0 ^b,c^	3.6 ^a^	3.9 ^b,c^	3.5 ^a^
Dairy products	Higher price	3.9 ^b^	4.3 ^a^	3.8 ^b^	4.1 ^a^
	Good taste	3.8 ^b^	4.6 ^a^	4.0 ^b^	4.5 ^a^
	Healthy	3.3 ^b^	4.2 ^a^	3.5 ^b^	4.3 ^a^
Environment	Negative influence on the environment	2.6 ^b^	4.4 ^a^	2.6 ^b^	4.3 ^a^
	Image landscape	2.8 ^c^	3.8 ^a^	2.5 ^d^	3.7 ^a^

Notes: Values in the same row and sub-table not sharing the same subscript are significantly different at *p* < 0.05 in the two-sided test of equality for column means. Tests are adjusted for all pairwise comparisons within a row of each innermost sub-table using the Bonferroni correction. ^a,b^ The probability that respondents in the group with ‘a’ gave higher/lower importance levels that respondents in the group with ‘b’ was significant (*p* < 0.05) for that particular aspect. ^b,c^ The probability that respondents in the group with ‘b’ gave higher/lower importance levels that respondents in the group with ‘c’ was significant (*p* < 0.05) for that particular aspect. ^b,d^ The probability that respondents in the group with ‘b’ gave higher/lower importance levels that respondents in the group with ‘d’ was significant (*p* < 0.05) for that particular aspect.

## Data Availability

The data are not publicly available. Reviewers and editors can obtain the data upon request.

## References

[1] Blanco-Penedo I., Ouweltjes W., Ofner-Schröck E., Brügemmann K., Emanuelson U. (2020). Symposium review: Animal welfare in free-walk systems in Europe. J. Dairy Sci..

[2] Galama P.J., Ouweltjes W., Endres M.I., Sprecher J.R., Leso L., Kuipers A., Klopčič M. (2020). Symposium review: Future of housing for dairy cattle. J. Dairy Sci..

[3] Leso L., Barbari M., Lopes M.A., Damasceno F.A., Galama P., Taraba J.L., Kuipers A. (2020). Invited review: Compost-bedded pack barns for dairy cows. J. Dairy Sci..

[4] Ferreira Ponciano Ferraz P., Araújo e Silva Ferraz G., Leso L., Klopčič M., Rossi G., Barbari M. (2020). Evaluation of the Physical Properties of Bedding Materials for Dairy Cattle Using Fuzzy Clustering Analysis. Animals.

[5] Megan E., Waldrop J.R. (2021). Consumer acceptance and willingness to pay for cow housing systems in eight European countries. Q Open.

[6] Klopčič M., Erjavec K., Waldrop M., Roosen J., Engel P., Galama P., Kuipers A. (2021). Consumers’ and Farmers’ Perceptions in Europe Regarding the Use of Composted Bedding Material from Cattle. Sustainability.

[7] Bewley J.M., Robertson L.M., Eckelkamp E.A. (2017). A 100-year Review: Lactating Dairy Cattle Housing Management. J. Dairy Sci..

[8] Breckler S.J. (1984). Empirical validation of affect, behavior, and cognition as distinct components of attitude. J. Personal. Soc. Psychol..

[9] Weinrich R., Kühl S., Zühlsdorf A., Spiller A. (2014). Consumer attitudes in Germany towards different dairy housing systems and their implications for the marketing of pasture raised milk. Int. Food Agribus. Man..

[10] Kühl S., Gauly S., Spiller A. (2019). Analysing public acceptance of four common husbandry systems for dairy cattle using a picture-based approach. Livest. Sci..

[11] Boogaard B.K., Oosting S.J., Bock B.B. (2008). Defining sustainability as a socio-cultural concept: Citizen panels visiting dairy farms in the Netherlands. Livest. Sci..

[12] Ellis K.A., Billington K., McNeil B., McKeegan D. (2009). Public opinion on UK milk marketing and dairy cow welfare. Anim. Welf..

[13] Cardoso C.S., Hötzel M.J., Weary D.M., Robbins J.A., von Keyserling M.A.G. (2016). Imagining the ideal dairy farm. J. Dairy Sci..

[14] Clark B., Stewart G.B., Panzone L.A. (2016). A Systematic Review of Public Attitudes, Perceptions and Behaviours Towards Production Diseases Associated with Farm Animal Welfare. J. Agric. Environ. Ethics.

[15] Wilson P., Lewis M., Ackroyd J. (2014). Farm Business Innovation, Cooperation and Performance.

[16] van der Ploeg D.J. (2020). Farmers’ upheaval, climate crisis and populism. J. Peasant Stud..

[17] Scoones I.M., Edelman S., Borras M., Hall R., Wolford W., White B. (2018). Emancipatory Rural Politics: Confronting Authoritarian Populism. J. Peasant Stud..

[18] van der Ploeg J.D., Barjolle D., Bruil J., Brunori G., Costa Madureira L.M., Dessein J., Drąg Z., Fink-Kessler A., Gasselin P., de Molina M.G. (2019). The economic potential of agroecology: Empirical evidence from Europe. J. Rural Stud..

[19] Beaver A., Ritter C., von Keyserlingk M.A.G. (2019). The Dairy Cattle Housing Dilemma: Natural Behavior Versus Animal Care. Vet. Clin. N. Am. Food Anim. Pract..

[20] Kiełbasa B., Pietrzak S., Ulén B. (2018). Sustainable agriculture: The study on farmers’ perception and practices regarding nutrient management and limiting losses. J. Water Land Dev..

[21] Benedičič J., Erjavec K., Klopčič M. (2022). Environmental sustainability: Farmers’ views of housing systems for cattle. Ital. J. Anim. Sci..

[22] Bergstra T.J., Hogeveen H., Stassen E. (2017). Attitudes of Different Stakeholders Toward Pig Husbandry: A Study to Determine Conflicting and Matching Attitudes Toward Animals, Humans and the Environment. Agric. Hum. Values.

[23] Driessen C. (2012). Farmers engaged in deliberative practices; An ethonographic exploration of the mosaic of concerns in livestock agriculture. J. Agric. Environ. Ethics.

[24] Hötzel M.J. (2016). Letter to the editor: Engaging (but not “educating”) the public in technology developments may contribute to a socially sustainable dairy industry. J. Dairy Sci..

[25] Breeding and Milking Systems on Farms. Chamber of Agriculture and Forestry of Slovenia. https://www.kgzs.si/novica/nacin-reje-in-sistemi-molze-na-kmetijah-2021-04-28.

[26] Boogaard B.K., Bock B.B., Oosting S.J., Wiskerke J.S.C., van der Zijpp A.J. (2011). Social Acceptance of Dairy Farming: The Ambivalence Between the Two Faces of Modernity. J. Agric. Environ. Ethics.

[27] The Most Modern Floor in the World. Kmečki Glas. https://klaranahtigal.kmeckiglas.com/post/596413/najsodobnejsa-tla-na-svetu.

[28] Pawlak H., Maksym P. (2018). Modelling assessment of farmers workload. Con. Res. Trend Agric. Eng..

[29] Boogaard B.K., Oosting S.J., Bock B.B. (2006). Elements of societal perception of farm animal welfare: A quantitative study in The Netherlands. Livest. Sci..

[30] Hyland J.J., Jones D.L., Parkhill K.A., Barnes A.P., Williams A.P. (2016). Farmers’ perceptions of climate change: Identifying types. Agric. Hum. Values.

[31] Kelemen E., Nguyen G., Gomiero T., Kovacs E., Choisis J., Choisis N., Paoletti V., Podmaniczky L., Ryschawy J., Sarthou J. (2013). Farmers’ perceptions of biodiversity: Lessons from a discourse-based deliberative valuation study. Land Use Policy.

[32] Verbeke W. (2009). Stakeholder, citizen and consumer interests in farm animal welfare. Anim. Wel..

[33] Kendall H.A., La Bao L.M., Sharp J.S. (2006). Public concern with animal well-being: Place, social structural location, and individual experience. Rural Sociol..

[34] (2019). Slovenian Agriculture in Numbers. Agricultural Institute of Slovenia. https://www.kis.si/f/docs/Slovensko_kmetijstvo_v_stevilkah_OEK/KIS_Slovensko_kmetijstvo_v_stevilkah_2019_SLO_splet_.pdf.

[35] Kurtzo F., Hansen M.J., Rucker K.J., Edgar L.D. (2016). Agricultural Communications: Perspectives from the Experts. J. Appl. Commun..

[36] Gaworski M., de Cacheleu C., Inghels C., Leurs L., Mazarguil C., Ringot B., Tzu-Chen C. (2021). The Topic of the Ideal Dairy Farm Can Inspire How to Assess Knowledge about Dairy Production Processes: A Case Study with Students and Their Contributions. Processes.

